# Predictors of gallstone composition in 1025 symptomatic gallstones from Northern Germany

**DOI:** 10.1186/1471-230X-6-36

**Published:** 2006-11-22

**Authors:** Clemens Schafmayer, Jürgen Hartleb, Jürgen Tepel, Stefan Albers, Sandra Freitag, Henry Völzke, Stephan Buch, Markus Seeger, Birgit Timm, Bernd Kremer, Ulrich R Fölsch, Fred Fändrich, Michael Krawczak, Stefan Schreiber, Jochen Hampe

**Affiliations:** 1 Department of General and Thoracic Surgery, University Hospital Schleswig-Holstein, Campus Kiel, Arnold-Heller-Str. 7, D-24105 Kiel, Germany; 2 Laboratory Arndt and Partner, Lademannbogen 61–63, Hamburg, D-22339 Hamburg, Germany; 3 Department of General Internal Medicine, University Hospital Schleswig-Holstein, Campus Kiel, Schittenhelmstr. 12, D-24105 Kiel, Germany; 4 Institute of Medical Statistics and Biometry, University Hospital Schleswig-Holstein, Campus Kiel, Brunswiker Str. 10, D-24105 Kiel, Germany; 5 Department of Community Medicine, University of Greifswald, Walter-Rathenau-Str. 48, D-17487 Greifswald, Germany; 6 POPGEN Biobank, University Hospital Schleswig-Holstein, Campus Kiel, Schittenhelmstr. 12, D-24105 Kiel, Germany; 7 Institute of Clinical Molecular Biology, University Hospital Schleswig-Holstein, Campus Kiel, Schittenhelmstr. 12, D-24105 Kiel, Germany

## Abstract

**Background:**

Gallstones represent a prevalent and costly health problem. The changing epidemiology and the emerging non-surgical interventions for gallstone disease necessitate the definition of target populations for future therapies. This study aimed to define patterns of gallstone composition and identify demographic predictors of gallstone composition in a large sample of symptomatic gallstones from Northern Germany.

**Methods:**

One thousand and seventy-four post-cholecystectomy gallstone specimens were obtained. Demographic and clinical information was provided by questionnaire (N = 1025 independent individuals with complete information). Two samples from each gallstone were analyzed using Fourier transformed infrared spectrometry.

**Results:**

The most prevalent substance was cholesterol, which was detected in 95.0% of gallstone specimens. Bilirubin and bilirubinate were present in 30.0% and calcium was detected in 10.0% of the spectra. Ninety-two percent of measurements from the same stone yielded the same "main" substances, indicating a homogenous stone composition in most cases. Female sex and higher body mass index (BMI) were associated with the presence of cholesterol as a main substance in the gallstones (p < 0.001).

**Conclusion:**

The changing epidemiology of gallstone disease is reflected by a marked shift in stone composition: Only two percent of stones in this study were pigment stones as compared to 91% percent of stones containing cholesterol as a main substance. Obese individuals from Germany with a BMI > 30 kg/m^2 ^have in 95% cholesterol-dominant gallstones and represent a potential target population for non-surgical interventions for the prevention or treatment of cholesterol stones.

## Background

Gallstones represent a serious burden for Western healthcare systems: 10–20% of Europeans and Americans carry gallbladder stones [1,2]. The prevalence of gallstone disease is rising, possibly as a result of longer life expectancy and altered nutritional habits. Many gallstones are silent, but symptoms and complications ensue in around 25–50% of cases, necessitating surgical removal of the gallbladder, usually by laparoscopic cholecystectomy [2,3]. Each year, more than 170,000 cholecystectomies are performed in Germany [4]. Cholelithiasis incurs annual medical expenses in excess of $6 billion in the US and is currently the second most expensive digestive disease, exceeded only by reflux disease [5]. The clinical management of gallstone disease is almost exclusively based on cholecystectomy and endoscopic or medical treatment of complications. Cholecystectomy, although an established procedure, still carries a small but existent complication rate, especially when performed in an acute setting. Mortality rates following cholecystectomy range from less than 0.1% in clinical studies to 0.7% (as documented for all cholecystectomies performed in Germany in 2004) [4]. In the US, about 3,000 deaths (0.12% of all deaths) per year are attributed to complications of cholelithiasis and gallbladder disease [6].

An improved etiological and pathophysiological understanding of gallstone disease may lead to novel, non-surgical, options for prevention and therapy. Clinically, high risk groups with small gallstones have recently been defined [7]. Significant progress has been made both in the genetics of gallstone formation [1,8] and in the molecular biology of bile excretion [9-11]. Consequently, this mechanistic knowledge may be lead to novel non-surgical therapeutic or preventive strategies, as for instance shown by the prevention of cholesterol gallstone formation in a mouse model by FXR agonists [12] or the cholesterol absorption inhibitor ezetimibe [13]. Gallstones, however, are heteroneous both in terms of their composition and their pathogenesis. The application of novel preventive or therapeutic approaches will likely be limited to certain classes of gallstones as defined by their composition and etiology. Therefore, in order to select the correct patient groups for such interventions, there is a need to idenitify clinical predictors of stone composition.

Gallstone disease is a disorder with a changing prevalence, reflecting the increasing life expectancy and changes in life style in "westernized" societies [1]. In particular, the increase of life style related risk factors was assumed to result primarily in an increase of cholesterol gallstones [14]. Gallstone composition, however, has not attracted adequate attention in current population-based studies leaving uncertainty regarding the confirmation of this assumption. The impact of life style and ethnicity is underscored by the profoundly different gallstone compositions in recent studies from sub-Saharan Africa [15] and China [16,17].

Classical chemical analysis of gallstone samples is a very laborious methodology. Fourier Transform Infrared (FTIR) spectroscopy has been established as a means of gallstone analysis by Japanese [18] and US investigators [19]. The quantitative reliability has been confirmed in systematic admixtures studies [19]. Thus, the utilization of FTIR makes the analysis of larger series of gallstones samples possible. We thus used FTIR, in order to define target populations for future therapeutic and preventive therapies and to investigate the possibly changing gallstone composition on a background of the rising prevalence. We obtained gallstone specimens from 1074 cholecystectomies from Northern Germany. We generated descriptive measures of stone composition in this population and investigated potential predictors of stone composition.

## Methods

### Patients and phenotypes

The 2001–2004 cholecystectomy statistics of Northern Schleswig-Holstein were extracted from the German National Quality Control data ("Qualitätssicherungsdaten Cholezystektomie"). Ten hospitals were found to have performed 93% of cholecystectomies in the region. All patients who had undergone cholecystectomy for gallstone disease during the study period, and who at or under the age of 65 years at the time of diagnosis, were contacted via the respective treatment unit by mail and offered participation in the study. One written reminder was sent to non-responders. Individuals who agreed to participate were interviewed by mail questionnaire. In most departments, it is customary to hand the removed gallstones to the patient. Participants were thus provided with dedicated containers and asked to either send or hand in their gallstones along with the questionnaire. Recruitment was performed through "popgen", a comprehensive regional biobank project in Northern Schleswig-Holstein [20], and utilised their technical, ethical and data protection protocols. All study protocols were approved by the institutional ethics committee ("Ethikkommission des Universitätsklinikums Schleswig-Holstein, Kiel) and by the public data protection agency. Written informed consent was obtained from all study participants. Additional patient samples were (N = 204) recruited at the Krankenhaus Lüneburg, a single centre outside Northern Schleswig-Holstein, located in Northern Germany approximately 200 kilometers from the popgen area. Recruitment was performed following the procedures described above.

In total, 9992 patients with operation records fulfilling the recruitment criteria were identified and contacted by mail. Out of these, 1539 patients could not be contacted, because they had either changed address or had died since the operation. Out of the remaining 8453 patients, 3174 patients participated in the overall study, i.e. provided questionnaires and a venous blood sample. Out of these, 1074 patients were able to provide a gallstone. Part of this are 87 consecutive patients, who were directly recruited in the surgical department of the Kiel university during 2005. Therefore, compared to the total operated population under or at the age of 65, 10.3% provided a gallstone sample and complete clinical information (N = 1025). This corresponds to 32.3% of the participating patients.

From all patients, information on the age at the time of operation, sex, height and weight, parity and a family history in the first-degree relatives was obtained by questionnaire. Eighty-nine percent of patients were ethnic Germans as defined by the birthplace of both parents.

### Analysis of gallstone composition

Dry gallstone specimens were fragmented using a scalpel. The weight of the gallstone was measured and two samples were obtained from each gallstone: The sites of sampling of an individual stone were chosen to be as different in macroscopic appearance as possible. For example, the pigmented core and the yellow shell of a stone would have been sampled if present. If multiple stones were available for one patient, the largest stone was investigated. The samples were ground separately with a pestle and mortar to a homogenous powder. One to two milligrams of the resulting product were mixed with 300 mg milligrams of potassium bromide (L.O.T.-Oriel, Darmstadt, Germany) and pressed with eight to nine tons (corresponding to 0.7 to 1 GPa) to generate a KBr disk with 13 mm diameter. These slices were measured using Fourier Transform Infrared (FTIR) spectrometry on a FTS 155 FT-IR spectrometer (Biorad, Hercules, Ca, USA) in a range of 400 to 4000 cm^-1 ^at 4 cm^-1 ^resolution. Control spectra from the manufactures library were used and additional spectra were obtained for analysis grade cholesterol, bilirubin and synthesized samples of calcium and magnesium bilirubinate. Stone composition was determined on the basis of the FTIR-spectra after analysis by the Biorad Win-IR software (version 2.04).

To facilitate further analysis, the composition of the gallstones was classified into "main", "intermediate" and "trace" components: Substances were assigned as "main" components if they constituted more than 30% of the gallstone (weight/weight). Components were classified as "trace" components if the respective substance constituted less then 10 percent (weight/weight) of the gallstone. Components in the range of 10 to 30% were classified as "intermediate". All bilirubinate salts were summarized as "bilirubin", i.e. the different bilirubin salts were not differentiated. A justification of this classification scheme is given in the discussion.

### Statistical analysis

Data was analyzed using the open source implementation of the S statistical language in the R software package and SPSS version 11. Contingency tables were analyzed through chisquared statistics or Fisher's exact test, as appropriate. Normality of distributions was assessed using a one sample Kolmogorov-Smirnov test. Nonparametric comparisons of non-normally distributed variables were performed using the Wilcoxon test.

## Results

### Principal types of gallstones

A total of 1074 gallstone samples were available for analysis. The chemical substances detected in each gallstone were classified as "main", "intermediate" or "trace" components as described in the Methods section. The justification for using this conservative classification rather than estimated percentages is given in the discussion. Table 1 provides a descriptive overview of the detection of the substances in the respective semiquantitative categories. Numbers in each of the columns do not add up to 100% because of the categorical classification schema. For instance, up to three "main" substances were detected in some gallstones (N = 5 with three "main" substances). If a particular substance was detected in a higher quantitative category, it was not listed in either of the other categories. Thus, the values in Table 1 can be for instance interpreted as: "how often was substance X detected as a trace substance in the stone samples?". The most prevalent substance was cholesterol, which was detected in 95.0% of gallstone specimens. Bilirubin was present in 30.0% and calcium was detected in 10.0% of the spectra. Rare components of gallstones included palmitate/stearate, polysaccharides and struvite, the being detected in one gallstone.

Based on the apparent diversity in the stone composition, two questions were asked: i) Are the substances given in Table 1 freely combined or do the stone samples fall into distinct categories?; and ii) to what extent are the two samples taken from the gallstones correlated – i. e. are stones homogenous or heterogeneous in composition? To answer the first question, the main and intermediate substances of the 2148 measurements were analyzed in a cross-table (Table 2). Indeed, the components are not freely combined, but the diversity of components reduces to few composition types (p < 0.001). The most common category is the dominant cholesterol stone (N = 1847) in 86% of analysed specimens. These stones would contain 70% or more cholesterol as no other "main" substance was detected. The next most common type of local composition is the combined cholesterol (as main substance) and bilirubin (as intermediate) setting (4.1%). Dominant calcium (1.0%) and dominant bilirubin (2.1%) compositions are both already quite uncommon. All other types of combinations of chemical substances were rare observations, with each less than 1% prevalence in the investigated specimens. Based on the data in Table 2, measurements were then classified. Class "C" denotes dominant cholesterol compositions (cholesterol as a main substance and no intermediate substances). The other categories were cholesterol – bilirubin (CB), bilirubin (B), calcium (CA) and other (O). The "O" category totally accounted for 7.4% of spectra.

Using this categorization, the second question was tackled. The correlation of the two spectra obtained from each gallstone was investigated in a cross-table (Table 3). This table is principally populated along the diagonal – indicating homogenous stone composition in most cases. Specifically, in 82.5% of stones, both spectra yielded a cholesterol composition. Interestingly, 0.2% of stones yielded a pure "B" and "C" classification and 5.2% of stones a "C" and "CB" classification. These stones correspond to the previously described pigmented centers or cholesterol stones with pigment layers [21]. Based on Table 3, a patient-oriented classification of stone composition was generated as indicated in this table into the "C", "CB", "B" and "O" categories. The composition of the gallstones from the 87 directly recruited consecutive patients from the Kiel surgical department did not differ significantly from the overall population (p > 0.2).

### Correlation of patient and stone characteristics

For an exploratory analysis, the median and interquartile ranges of the patient and stone characteristics were tabulated by stone classification (Table 4). For this analysis, stones from relatives and patients with incomplete demographic information were excluded yielding N = 1025 patients with a classified gallstone. None of the quantitative measures followed a normal distribution (Komolgorov-Smirnov-tests for goodness of fit p < 0.05). Thus medians and interquartile ranges are used in the table and non-parametric statistics were used for group comparisons. Interestingly, the "C" and "CB" classes of are very similar in their characteristics (p > 0.05). The sex distribution between these two categories was, however, different with 23% versus 38% males in the respective categories (χ^2 ^= 9.1, df = 1, p = 0.002). The stones that contained more bilirubin than cholesterol (N = 12) but both as main component (i.e. > 30% cholesterol), which were classified as "CB" accounted for this difference. Overall, the most striking differences were observed between the pigment stones (B) on one hand and predominant cholesterol stones (C and CB) on the other hand. The following results refer to the comparison of these two groups: Pigment stones are more prevalent in males (58%) than in females (χ^2 ^= 12.6, df = 1, p = 0.0008), and stones are smaller with a median weight of 0.6 grams (Z = -3.7, p < 0.001). The age at operation was marginally significant (Z=-2.0, p = 0.045), between the groups possibly due to the lack of paediatric patients in the present sample. The parity and differences in reported family history are not statistically significant (p > 0.05). To further investigate the difference in BMI between the pigment and cholesterol stones, the prevalence of the combined C and CB stones for different categories of BMIs are presented in Table 5. Patients with lower BMIs have a lower prevalence of cholesterol stones (χ^2 ^= 17.0, df = 3, p = 0.0007). In the subgroup analysis, this was significant in females (χ^2 ^= 16.5, df = 3, p = 0.0009), and males (Fishers exact test point p-value = 0.0003). The "other" stone category shares many similarities in patient characteristics with the cholesterol stones (Table 4). Due to the small number and heterogeneous composition of this group of stones, epidemiological predictors were not explored in further detail.

As an illustration of the different stone types, a photograph of representatives of the respective groups is provided [see Additional file 1].

## Discussion

Fourier Transform Infrared (FTIR) spectroscopy has been established as a means to the rapid assessment of the composition of gallstones [18,19]. Only this methodology enabled the analysis of the over 1000 stone samples and was thus used to investigate this large sample of gallstones. We have chosen to present the results of the FTIR in a semi-quantitative classification rather than exact numbers of the proportion of the respective stone components, because the direct quantification of components using FTIR is limited by some interfering factors: Namely, interaction of the components with the KBr, differences in hydration, ion exchange reactions, the degree of dispersion between KBr and gallstone components, the Christiansen-effect and/or possible chemical reactions of components can influence the relative height and appearance of the spectrum peaks [22-24]. Thus, in order to obtain robust (and conservative) estimates of gallstone composition, the substances were classified into "main", "intermediate" and "trace" components (as described in the Methods section) rather than being expressed in explicit percentages.

The fine structure [21,25], bacterial colonization [26] and mechanistics of gallstone generation [27-30] have been studied in detail in previous experiments. We think, that this study still makes a substantial contribution in that it has focussed more on a general epidemiological survey of gallstone composition mainly to determine the principal components of the current gallstones in order to help identify patients for future innovative non-surgical interventions [12]: In the descriptive statistics of gallstone composition, two main conclusions can be drawn: Firstly, gallstones in the investigated population are quite homogenous in terms of their main components: In only eightytwo (7.6%, including sixty stones, that contained bilirubin/cholesterol combinations) out of 1074 stones, different main substances were identified in the two samples taken from each stone (Table 3). Most of this relates to the inclusion of bilirubin in otherwise dominant cholesterol stones, thus representing the pigment cores or rings in cholesterol gallstones, which have been studied previously in detail [21]. The other source of heterogeneity stems from calcium as an admixture to gallstones [25,31]. The quantitatively major components of gallstones identified in this experiments are cholesterol, bilirubin (pigment) [32] and calcium carbonate [31] with 93.3%, 5.5% and 4.8% relative frequencies of detection as main substances (Table 2). These major components are not randomly assembled in gallstones (p < 0.001), but the composition falls into distinct classes of gallstones, namely cholesterol, bilirubin (pigment) and calcium stones. This classification has obviously been developed before and is here confirmed on a more formal level. Thus, for further analysis, stones were classified into four classes (for exact definition see the Results section). Calcium was detected only in 10% of gallstones, which has implication for the sonographic and radiographic detection of these concrements.

Interestingly, the age of onset was not strongly correlated to stone composition (p = 0.045), this is probably due to the lack of coverage of the paediatric population in this survey: The youngest patients in this study were 18 years at diagnosis. Thus, the reported very different gallstone composition of approximately 50% pigment stones and 35% calcium stones in children [33,34] was not observed here.

As a main result of the search for epidemiological predictors of stone composition, sex and BMI were identified: Both associations were significant (p < 0.001). Interestingly, in obese individuals (BMI≥30), approximately 95% of gallstones consist predominantly of cholesterol for both men and women. Thus, the increase in gallstone prevalence and the association of overall gallstone risk with higher BMI [2] are likely resulting primarily in an increase of cholesterol gallstones.

The study has addressed a selected patient population and is thus not population-representative: This applies to the focus on younger patients who were operated at an age under 65, the overall response rate as compared to the total operated population of (10.3%) and to potential bias introduced through storage of gallstone samples by the patients. Here, the personality traits of the patients (orderliness etc.) and the stability of stones may have played a role. Stones that tend to disintegrate are more likely to be of rarer composition types (polysaccharide, certain calcium stones) and may have been thus underrepresented in this investigation. The overall pattern of stone composition was similar in the 87 consecutive patients directly recruited at the Kiel university hospital in 2005 thus indicating, that main findings may robust against these potential selection bias factors.

Taken the limitations noted above into account, the study shows the changing patterns of gallstone disease and confirms the need for the present investigation. In this experiment cholesterol stones represent the by far dominating type of gallstone. In contrast, studies from the 1960is and 1970ies have shown prevalences of pigment stones of 23 [19] to 30 percent [35]. In our study, pigment stones are relatively infrequent with only two percent of stones in the present sample of symptomatic gallstones. Previous studies from Western countries have also shown a predominance of cholesterol stones with 100% of patients under the age of 50 and 60% for older patients [36] in a Dutch study and 58% of cholesterol stones in a U.S. study using visual inspection of stones [19]. If the definition of a cholesterol stone from van Erpecum et al. is adapted to our data, 91.1% of stones contain 70% or more cholesterol (row 1 of Table 2). Some 2.2% of stones contain between 30 and 70% cholesterol and cannot be clearly by put into the 50% categorisation (rows 2–3 of Table 2). Although no correlation of age of operation and stone composition was found, this might be due to the exclusion of old patients with gallstones. Thus, the high proportion of cholesterol gallstones in this investigation may be due to this age selection and also related to the same altered nutritional patterns that also lead to an increase in gallstone prevalence in susceptible populations.

## Conclusion

In summary, this study documents a dramatic change in gallstone composition – in the high-prevalence region of Northern Germany – with a overwhelming preponderance of cholesterol-based gallstones. This is in contrast to the gallstone composition in recent studies from sub-Saharan Africa [15] and China [16,17]. Based on the findings of this study, a potential homogenous target population in Germany for non-surgical intervention [12,13] for the prevention of cholesterol stones would be obese individuals with a BMI > 30, for which approximately 95% of cholesterol gallstones were observed in both males and females.

## Competing interests

The author(s) declare that they have no competing interests.

## Authors' contributions

CS, JT, BT, MS, SB recruited and characterized the patients. SF, MK, JH and HV performed and supervised the data analysis. JüH and SA performed the FTIR analysis of stone composition. BK, URF, FF and SS participated in study design and the drafting of the manuscript. MK and SS structured the patient recruitment project. JH conceived and coordinated the study and drafted the manuscript together with all other coauthors.

## Pre-publication history

The pre-publication history for this paper can be accessed here:



## Supplementary Material

Additional file 1 Examples of gallstones and their chemical composition. The figure provides examples of the analyzed gallstones and their chemical composition. Click here for file

## Figures and Tables

**Table 1:** General descriptive overview of the composition of the gallstone samples:

**Substance**	**Main** **(> 30%)**	**Intermediate** **(10–30%)**	**Trace** **(< 10%)**	**Qualitative presence**
***Cholesterol***	93.3%	0.8%	0.9%	95.0%
***Bilirubin***	5.5%	4.7%	19.8%	30.0%
***Calcium (any mineral)***				10.0%
Ca_5_(PO_4_)_3_(F,Cl,OH) – Apatite	1.2%	0.4%	1.5%	3.1%
CaCO_3 _– Aragonite	1.3%	0.1%	0.1%	1.5%
CaCO_3 _– Calcite	1.0%	0.4%	0.3%	1.7%
CaCO_3 _– mineral not defined	1.3%	0.7%	1.7%	3.7%
***Magnesium***				
Struvite (NH_4_)MgPO_4_·6H_2_O	Not observed	0.1%	Not observed	0.1%
***Palmitate/Stearate***	0.1%	0.3%	0.2%	0.7%
***Polysaccharide***	0.1%	0.2%	0.3%	0.6%

The frequency of detection of either of the substances in the 2148 FTIR spectra obtained from the 1074 gallstones is given in the left column for the quantitative classifications of "Main", "Intermediate" and "Trace" components. The term "Qualitative presence" refers to the detection of the respective substance in any of the categories. Note, that numbers will not sum to 100% due to presence of multiple stone components. All bilirubinate salts were summarized as "bilirubin", i.e. the different bilirubin salts were not differentiated.

**Table 2:** Principal stone types:

		Intermediate substances																
			
Main substances		none	Apatite	Aragonite	Bilirubin	Bilirubin Apatite	Chol.	Chol. Calcite	Chol. Palmitate	Ca	Ca Apatite	Ca Bilirubin	Calcite	Calcite Bilirubin	Palmitate	Struvite	Polysaccharide	
Cholesterol		***0.860***	0.001		*0.041*	0.001				0.006		< 0.001	0.002					0.911
Cholesterol	Bilirubin	*0.013*	< 0.001												< 0.001			0.014
Cholesterol	Bilirubin, Apatite	0.003																0.003
Cholesterol	Calcium	0.004			0.001													0.005
Bilirubin		***0.021***	0.001	< 0.001			0.002		0.001		< 0.001				0.001			0.027
Bilirubin	Apatite	0.005						0.001		< 0.001								0.006
Bilirubin	Aragonite	0.003																0.003
Bilirubin	Calcium	0.001																0.001
Apatite					< 0.001									< 0.001				0.001
Apatite	Colesterol				< 0.001													< 0.001
Apatite	Calcium	< 0.001																< 0.001
Aragonite		0.006					0.003											0.009
Aragonite	Colesterol				< 0.001													< 0.001
Calcium		0.002			0.002		0.001										0.001	0.006
Calcite		0.007														0.001		0.008
Calcite	Apatite	0.001																0.001
Calcite	Cholesterol	0.001																0.001
Stearate		0.001																0.001
Polysaccharide																	0.001	0.001

																		**1**

The main and intermediate substances from all 2148 FTIR measurements are displayed in this cross-table. It is evident, that the various stone components are not mixed at random but rather fall into few, specific categories. Cells with "pure" stones and a frequency above 1% are marked in bold print. The combination of cholesterol and bilirubin (two cells) is marked in underlined italics. Values of "< 0.001" correspond to single observations. Abbreviations in the Table heading: Chol. Corresponds to Cholesterol and Ca corresponds to Calcium.

**Table 3:** Comparison of the two spectra obtained from the same stone:

	Spectrum B			
	
Spectrum A	C	B	CB	O
C	0.825			
B	*0.002*	0.019		
CB	*0.052*	*0.002*	*0.027*	
O	***0.016***	***0.001***	***0.004***	***0.053***

Two FTIR spectra were obtained from each gallstone from two sites chosen to be macroscopically as different as possible. The symmetrical fields on the upper right hand corner of the table were collapsed into the triangular shape for ease of interpretation. It is evident, that stones are quite homogenous with 92.4 percent of stones yielding the same classification in both measurements. Based on this table, gallstones were classified for the following patient-oriented analyses as "C" (cholesterol), "B" (bilirubin), "CB" (cholesterol – bilirubin: underlined italics in the table) and "O" (other – bold italics).

**Table 4:** Exploratory analysis of stone composition in 1025 unrelated index patients.

	B	C	CB	O
N	19	846	85	75
stone weight	0.6 (0.2–1.1) g	1.6 (0.7–3.4) g	2.0 (1.0–4.9) g	0.7 (0.2 – 1.5) g
%male	58%	23%	38%	28%
BMI	25.5 (23.1 – 29.1)	27.1 (24.7 – 30.4)	27.1 (25.0 – 29.1)	27.0 (23.7 – 29.0)
parity (females)	2 (2 – 2)	2 (1 – 3)	2 (1 – 2)	2 (2 – 2)
age of operation	60 (48 – 63)	53 (43–60)	56 (44–62)	55 (47 – 61)
family history	53%	46%	45%	36%

For quantitative measures (stone weight, BMI, parity, age of operation), the median and interquartile range are reported. Other measures are expressed as percentages. The family history refers to a positive family history of gallstones (either history of cholecystectomy or self-reported presence of gallstone based on previous sonography) in a first degree relative.

**Table 5:** Joint presence of the "C" and "CB" stone compositions in relation to sex and BMI.

Sex	BMI			
	
	0–20	20–25	25–30	> 30
	
***All***	***74%***	***89%***	***91%***	***95%***
female	72%	91%	92%	94%
male	83%	79%	88%	96%

The frequency of this composite type of gallstone in the respective age and BMI categories is reported.

## References

[B1] Lammert F, Sauerbruch T (2005). Mechanisms of disease: the genetic epidemiology of gallbladder stones. Nat Clin Pract Gastroenterol Hepatol.

[B2] Volzke H, Baumeister SE, Alte D, Hoffmann W, Schwahn C, Simon P, John U, Lerch MM (2005). Independent risk factors for gallstone formation in a region with high cholelithiasis prevalence. Digestion.

[B3] Ransohoff DF, Gracie WA (1993). Treatment of gallstones. Ann Intern Med.

[B4] BQS Bundesgeschäftsstelle Qualitätssicherung, BQS Bundesgeschäftsstelle Qualitätssicherung (2004). Cholezystektomie. Qualität sichtbar machen BQS-Qualitätsreport 2004.

[B5] Sandler RS, Everhart JE, Donowitz M, Adams E, Cronin K, Goodman C, Gemmen E, Shah S, Avdic A, Rubin R (2002). The burden of selected digestive diseases in the United States. Gastroenterology.

[B6] U.S. Department of Health and Human Services, National Institutes of Health (2005). Gallbladder and biliary disease. Action plan for liver disease research A report of the Liver Diseases Subcommittee of the Digestive Diseases Interagency Coordinating Committee.

[B7] Venneman NG, Renooij W, Rehfeld JF, van Berge-Henegouwen GP, Go PMNYH, Broeders IAMJ, van Erpecum KJ (2005). Small Gallstones, Preserved Gallbladder Motility, and Fast Crystallization Are Associated with Pancreatitis. Hepatology.

[B8] Wang DQ, Afdhal NH (2004). Genetic analysis of cholesterol gallstone formation: searching for Lith (gallstone) genes. Curr Gastroenterol Rep.

[B9] Trauner M, Boyer JL (2003). Bile salt transporters: molecular characterization, function, and regulation. Physiol Rev.

[B10] Kullak-Ublick GA, Stieger B, Meier PJ (2004). Enterohepatic bile salt transporters in normal physiology and liver disease. Gastroenterology.

[B11] Yu L, Gupta S, Xu F, Liverman AD, Moschetta A, Mangelsdorf DJ, Repa JJ, Hobbs HH, Cohen JC (2005). Expression of ABCG5 and ABCG8 is required for regulation of biliary cholesterol secretion. J Biol Chem.

[B12] Moschetta A, Bookout AL, Mangelsdorf DJ (2004). Prevention of cholesterol gallstone disease by FXR agonists in a mouse model. Nat Med.

[B13] Wang HH, Portinacase P, Wang DQ (2006). Prevention of Cholesterol (Ch) Gallstones by the Potent Ch Absorption Inhibitor Ezetimibe in Gallstone-Susceptible Mice. Gastroenterology.

[B14] Paigen B, Carey MC, King RA (2002). Gallstones. The genetic basis of common diseases.

[B15] Angwafo FF, Takongmo S, Griffith D (2004). Determination of chemical composition of gall bladder stones: basis for treatment strategies in patients from Yaounde, Cameroon. World J Gastroenterol.

[B16] Ho KJ, Lin XZ, Yu SC, Chen JS, Wu CZ (1995). Cholelithiasis in Taiwan. Gallstone characteristics, surgical incidence, bile lipid composition, and role of beta-glucuronidase. Dig Dis Sci.

[B17] Tsai WL, Lai KH, Lin CK, Chan HH, Lo CC, Hsu PI, Chen WC, Cheng JS, Lo GH (2005). Composition of common bile duct stones in Chinese patients during and after endoscopic sphincterotomy. World J Gastroenterol.

[B18] Chihara G, Yamamoto S, Kameda H (1958). Medical and biochemical application of infrared absorption spectra. I. Studies on gallstone by infrared spectra and X-ray chrystallography. Chem Pharm Bull (Tokyo).

[B19] Trotman BW, Ostrow JD, Soloway RD (1974). Pigment vs cholesterol cholelithiasis: comparison of stone and bile composition. Am J Dig Dis.

[B20] Krawczak M, Nikolaus S, von Eberstein H, El Mokhtari NE, Schreiber S (2006). PopGen: Population-based recruitment of patients and controls for the analysis of complex genotype-phenotype relationships. Community Genet.

[B21] Malet PF, Williamson CE, Trotman BW, Soloway RD (1986). Composition of pigmented centers of cholesterol gallstones. Hepatology.

[B22] Milne JW (1976). Ion exchange in alkali halide disks. Spectrochim Acta.

[B23] Ataman OY, Mark HB (1977). Alkali Halide Pelleting Technique for Solid Sampling in Infrared Spectroscopy. Appl Spectroscopy Rev.

[B24] Keresztury G, Insze M, Seti F, Imre L (1980). CO2 inclusion bands in infrared spectra of KBr pellets. Spectrochim Acta.

[B25] Malet PF, Weston NE, Trotman BW, Soloway RD (1985). Cyclic deposition of calcium salts during growth of cholesterol gallstones. Scan Electron Microsc.

[B26] Swidsinski A, Khilkin M, Pahlig H, Swidsinski S, Priem F (1998). Time dependent changes in the concentration and type of bacterial sequences found in cholesterol gallstones. Hepatology.

[B27] Gustafsson U, Sahlin S, Einarsson C (2003). High level of deoxycholic acid in human bile does not promote cholesterol gallstone formation. World J Gastroenterol.

[B28] Gustafsson U, Benthin L, Granstrom L, Groen AK, Sahlin S, Einarsson C (2005). Changes in gallbladder bile composition and crystal detection time in morbidly obese subjects after bariatric surgery. Hepatology.

[B29] Venneman NG, Portincasa P, Vanberge-Henegouwen GP, van Erpecum KJ (2004). Cholesterol saturation rather than phospholipid/bile salt ratio or protein content affects crystallization sequences in human gallbladder bile. Eur J Clin Invest.

[B30] Yago MD, Gonzalez V, Serrano P, Calpena R, Martinez MA, Martinez-Victoria E, Manas M (2005). Effect of the type of dietary fat on biliary lipid composition and bile lithogenicity in humans with cholesterol gallstone disease. Nutrition.

[B31] Taylor DR, Crowther RS, Cozart JC, Sharrock P, Wu J, Soloway RD (1995). Calcium Carbonate in Cholesterol Gallstones: Polymorphism, Distribution and Hypothesis About Pathogenesis. Hepatology.

[B32] Trotman BW, Soloway RD (1982). Pigment Gallstone Disease: Summary of the National Institutes of Health - International Workshop. Hepatology.

[B33] Stringer MD, Taylor DR, Soloway RD (2003). Gallstone composition: are children different?. J Pediatr.

[B34] Kleiner O, Ramesh J, Huleihel M, Cohen B, Kantarovich K, Levi C, Polyak B, Marks RS, Mordehai J, Cohen Z, Mordechai S (2002). A comparative study of gallstones from children and adults using FTIR spectroscopy and fluorescence microscopy. BMC Gastroenterol.

[B35] Friedman GD, Kannel WB, Dawler TR (1966). The epidemiology of gallstone disease: Observations in the Framingham study. J Chronic Dis.

[B36] van Erpecum KJ, van Berge Henegouwen GP, Stoelwinder B, Stolk MF, Eggink WF, Govaert WH (1988). Cholesterol and pigment gallstone disease: comparison of the reliability of three bile tests for differentiation between the two stone types. Scand J Gastroenterol.

