# Supplementary material for: Predictors of gallstone composition in 1025 symptomatic gallstones from Northern Germany
Source: BMC Gastroenterol. 2006 Nov 22;6:36. doi: 10.1186/1471-230X-6-36 (PMC1664574; doi:10.1186/1471-230X-6-36)

The following list provides the composition of the depicted stones.

	Spectrum A			Spectrum B		
Number	Main	Intermediate	Trace	Main	Intermediate	trace
1	Cholesterol	Bilirubin		Cholesterol		
2	Cholesterol			Cholesterol		Bilirubin
3	Cholesterol			Cholesterol		
4	Cholesterol		Bilirubin, Apatite	Cholesterol	Bilirubin	Apatite
5	Cholesterol			Cholesterol		
6	Cholesterol			Cholesterol		
7	Cholesterol			Cholesterol		
8	Cholesterol		Bilirubin	Cholesterol		Bilirubin
9	Cholesterol			Cholesterol		
10	Cholesterol			Cholesterol		
11	CaCO3			Cholesterol		
12	Polysaccharide			Polysaccharide		
13	Cholesterol			Cholesterol		
14	Aragonite			Aragonite		
15	Cholesterol			Cholesterol		
16	Bilirubin		Cholesterol	Bilirubin		Cholesterol
17	Aragonite		Bilirubin	Aragonite		Bilirubin
18	Cholesterol	Bilirubin		Cholesterol	Bilirubin	

Supplemental Figure 1

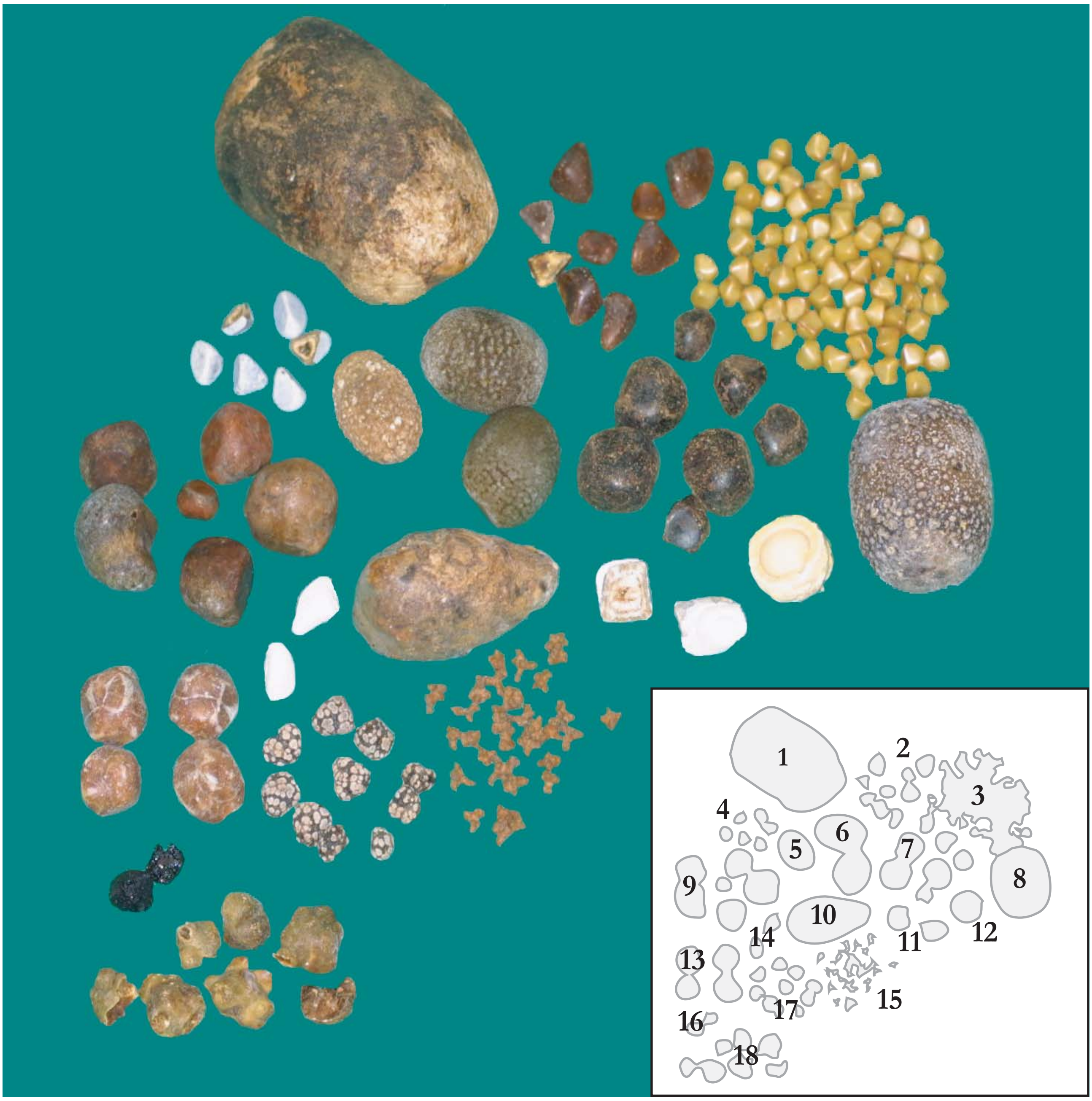

Supplement: Additional file 1 — Examples of gallstones and their chemical composition. The figure provides examples of the analyzed gallstones and their chemical composition. [file 1471-230X-6-36-S1.pdf]
